# Atrial Fibrillation Recurrence Risk After Catheter Ablation in Patients With Rheumatoid Arthritis: A Systematic Review and Meta‐Analysis

**DOI:** 10.1002/clc.70021

**Published:** 2025-01-16

**Authors:** Pongprueth Rujirachun, Phuuwadith Wattanachayakul, Svita Taveeamornrat, Patompong Ungprasert, Nithi Tokavanich, Krit Jongnarangsin

**Affiliations:** ^1^ Department of Medicine, Faculty of Medicine, Siriraj Hospital Mahidol University Bangkok Thailand; ^2^ Department of Medicine Albert Einstein Healthcare Network Philadelphia Pennsylvania USA; ^3^ Sidney Kimmel Medical College Thomas Jefferson University Philadelphia Pennsylvania USA; ^4^ Faculty of Medicine, Siriraj Hospital Mahidol University Bangkok Thailand; ^5^ Department of Rheumatic and Immunologic Diseases Cleveland Clinic Cleveland Ohio USA; ^6^ Department of Internal Medicine, Division of Cardiovascular Medicine University of Michigan Medical School Ann Arbor Michigan USA

**Keywords:** atrial fibrillation, catheter ablation, meta‐analysis, pulmonary vein isolation, recurrence, rheumatoid arthritis

## Abstract

**Background:**

The association between rheumatoid arthritis (RA) and the risk of developing atrial fibrillation (AF) is well‐established. However, data on the impact of RA on AF recurrence postcatheter ablation (CA) remain unclear. This current study aimed to assess the impact of RA on AF recurrence after catheter‐based pulmonary vein isolation.

**Methods:**

Potentially eligible studies were identified from Medline and EMBASE databases from inception to December 20, 2023. Eligible study must consist of two cohorts of patients with and without RA who underwent catheter ablation for AF. Pooled risk ratio (RR) and 95% CI were calculated using Dersimonian and Laird's random‐effect, generic inverse variance approach.

**Results:**

The meta‐analysis includes three retrospective cohort studies with a total of 700 patients. The pooled analysis found a significantly increased risk of AF recurrence after CA among patients with RA compared to patients without RA with the pooled RR of 1.59 (95% CI, 1.10–2.29, *I* ^2^ 14%). Increased risk of early recurrence (within 90 days) was also observed with the pooled RR of 2.70 (95% CI, 1.74–4.19, *I* ^2^ 0%).

**Conclusions:**

The current study found that patients with RA have a higher risk of AF recurrence after CA for AF, including the risk of early recurrence.

## Graphical abstract

The impact of RA on AF recurrence postcatheter ablation (CA) is unclear. Meta‐analysis of three cohort studies from Medline and EMBASE databases‐AF patients with or without RA undergoing CA for AF (700 patients). Patients with RA have a significantly higher risk of AF recurrence post‐CA.

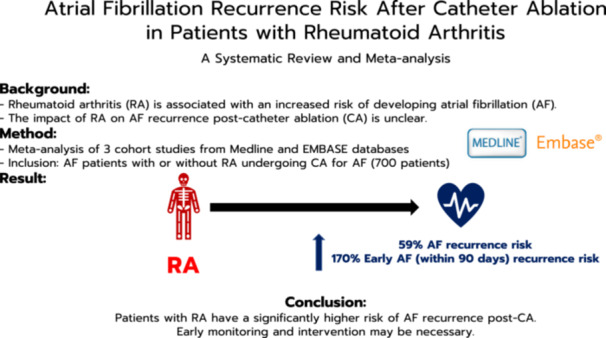

## 1 Introduction

Rheumatoid arthritis (RA) is an autoimmune disease characterized by the presence of inflammatory arthritis as well as extraarticular systemic inflammation. RA is one of the most common autoimmune diseases, impacting 0.25%–1% of the global population. It primarily affecting females, who constitute over two‐thirds of the patient demographic [1]. A significant proportion of women diagnosed with atrial fibrillation (AF) have an associated autoimmune condition [2]. Furthermore, individuals with RA exhibit a 29% higher occurrence of AF compared to general population [3]. The association between accelerated cardiovascular disease (CVD) and RA is also well‐established, with a 48% higher risk of CVD and a 50% elevated risk of CVD‐related mortality in RA patients [4].

AF is the most common cardiac arrhythmia that affects approximately 0.5% of the global population. It is characterized by atrial excitation at high frequencies, resulting in dyssynchronous atrial contraction and irregular ventricular excitation. AF is associated with reduced quality of life, increased mortality, and various cardiovascular conditions such as ischemic stroke, coronary artery disease, and heart failure [5, 6].

In addition, RA patients face challenges using antiarrhythmic drugs (AAD) for AF treatment due to adverse effects, reduced effectiveness, and specific contraindications associated with this condition [7]. Alternative treatment is catheter ablation (CA) with pulmonary vein isolation (PVI), which is effective for treatment of AF with or without structural heart disease [8].

Studies [9, 10, 11] have shown that CA is a safe procedure with a decent success rate for patients RA. However, data on the risk recurrence of atrial tachyarrhythmia after CA is conflicting. Some studies reported higher rate of early recurrence, increased use of AADs, and a higher likelihood of repeat ablation in RA patients [9, 10] but another study found a similar rate between RA patients and the general population [11]. In this meta‐analysis, we aimed to identify all available studies and summarize all available data to assess impact of RA on the risk of AF recurrence after catheter‐based pulmonary vein isolation.

## 2 Methods

### 2.1 Literature Search Strategy

Three investigators (P.R., P.W., and S.T.) independently searched for published literature indexed in the MEDLINE and EMBASE databases from the time of the study's inception to December 20, 2023. Their search technique included the keywords “CA” “AF” and “RA.” The search strategy is attached as Supporting Information S1: Data S1. A manual review of bibliography of selected articles was also performed to identify additional eligible studies. This study was conducted using the Preferred Reporting Items for Systematic Reviews and Meta‐Analyses (PRISMA) statement, which is available as Supporting Information S1: Data S2.

### 2.2 Selection Criteria

For a study to be included in the meta‐analysis, it must be either a cohort study or randomized controlled study that directly investigates the relationship between RA and the risk of AF recurrence after CA. Eligible study must consist of two cohorts, (1) patients with RA who underwent CA for AF and (2) patients without RA who underwent CA for AF. The study must report the rate of AF recurrence after the procedure in both groups. Recurrence within 3 months (90 days) is considered early recurrence, whereas any recurrence beyond that period is considered late recurrence. Eligible study must provide point estimate (relative risk [RR] or hazard ratio [HR]) and its corresponding 95% confidence interval (CI) to compare the risk between the two cohorts (or sufficient data to calculate the point estimate).

All retrieved articles were independently reviewed by the first three investigators (P.R., P.W., and S.T.) to determine eligibility. Different determination of eligibility was resolved by conference with all investigators. The Newcastle–Ottawa quality assessment scale [12] was used to evaluate quality of the included cohort studies. This scale evaluates the quality of the included studies in three domains: identification of the intended results, group comparability, and participant recruitment.

### 2.3 Data Extraction

The following data were extracted using a standardized data collection form: the first author's last name, study design, year(s) of the study, country of origin, year of publication, sample size, baseline characteristics of included patients, ablation strategies for AF, methods used to determine and confirm the diagnosis of AF, rate of AF recurrence and RA, adjusted confounders, and adjusted effect estimates with 95% confidence interval. Three investigators (P.R., P.W., and S.T.) independently performed this data extraction. Data collection forms were cross‐checked to ensure accuracy.

### 2.4 Statistical Analysis

The data were analyzed using the Cochrane Collaboration's Review Manager 5.3 software. The point estimates and standard errors from individual studies were extracted and pooled together to calculate pooled estimate using the generic inverse variance method as described by DerSimonian and Laird [13]. Given the difference in methodology and background population across the included studies, random‐effect model was used rather than fixed‐effect model. Both RR and HR were used as point estimate to calculate pooled risk ratio.

Cochran's *Q* test was performed to assess statistical heterogeneity. This statistic is supplemented by the *I* ^2^ statistic, which estimates the proportion of overall variation across studies that is due to heterogeneity rather than chance. Insignificant heterogeneity is represented by an *I* ^2^ value of 0%–25%, low heterogeneity is represented by a value of 26%–50%, moderate heterogeneity is represented by a value of 51%–75%, and high heterogeneity is represented by a value of > 75% [14]. If sufficient number of studies are included for the meta‐analysis, the presence of publication bias will be evaluated through the visualization of funnel plot.

## 3 Results

**Figure 1:**
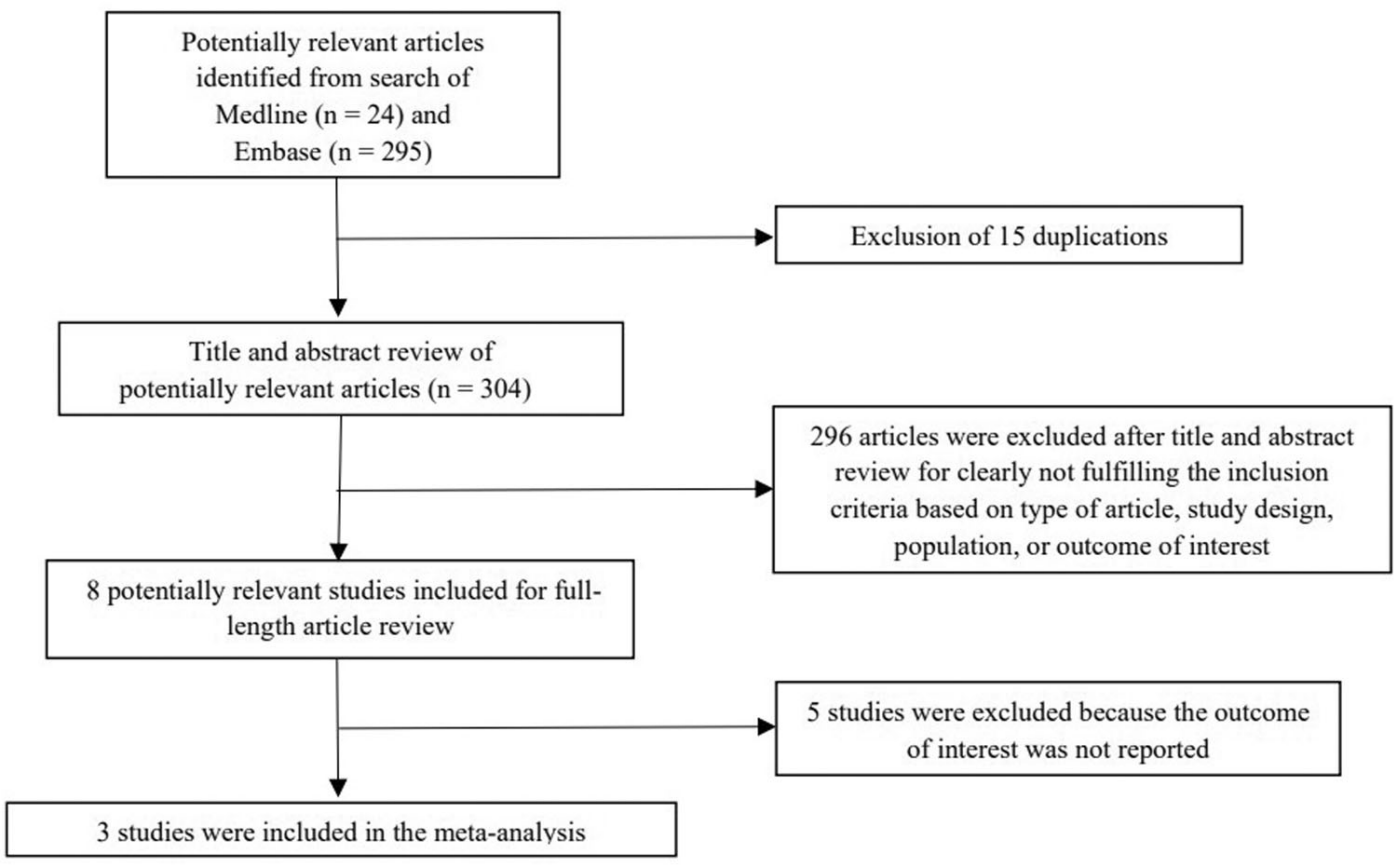
Flow‐chart of the literature review process.

The systematic search identified 319 relevant articles (295 articles from EMBASE and 24 articles from MEDLINE). After the exclusion of 15 duplicated articles, 304 articles underwent title and abstract review. A total of 296 articles were excluded at this stage as they did not fulfill the eligibility criteria based on the type of article, study design, participants, and outcome of interest. A total of eight articles were retrieved for full‐length article review and five articles were excluded at this stage as they did not report to the association of interest. Finally, three cohort studies [9, 10, 11] with 700 patients undergoing CA for AF (101 patients had RA) were eligible for the meta‐analysis. The literature retrieval, review, and selection process are shown in Figure 1. The characteristics of the included studies and their quality assessment are described in Table 1. AF recurrence was defined and assessed differently across the included studies. Most studies defined recurrence as any documented episode of AF lasting longer than 30 s, detected by electrocardiogram (ECG), Holter monitoring, or device interrogation. No blanking period was applied.

**Table 1:** Baseline characteristics of studies included in the meta‐analysis.

	Gao et al. [10]	Haq et al. [9]	Wen et al. [11]
Year of publication	2023	2022	2015
Country of origin	China	United States	China
Study design	Retrospective cohort study	Retrospective cohort study	Retrospective cohort study
Study subjects	Cases: Cases were patients who underwent CA for AF with a confirmed diagnosis of AD (RA is the most common AD) at the Beijing Anzhen Hospital and were screened from the China Atrial Fibrillation Registry. Cases were identified from 2012 to 2021 ChiCTR‐OCH‐13003729. Comparators: Comparators were the rest of the patients in the study who did not carry a diagnosis of AD. Median follow‐up time was 36.3 (11.8–60.5) months. Patients with previous ablation history for AF, valvular disease, previous cardiac surgery, congenital heart disease, cardiomyopathy, advanced HF (NYHA Class III–IV), and who were diagnosed with AD after the CA procedure were excluded from the analysis.	Cases: Cases were patients with RA undergoing CA for AF who were diagnosed based on the 2010 American College of Rheumatology/European League Against Rheumatism Collaborative Initiative. Cases were identified from 2010 to 2021 Mayo Clinic. Comparators: Comparators were the rest of the patients in the study who did not carry a diagnosis of RA. Follow‐up time was 1 year. Patients with a history of RA after catheter ablation were excluded from the analysis.	Cases: Cases were patients with RA who underwent CA for AF and were diagnosed based on the American Rheumatism Association Guidelines. Cases were identified from 2008 to 2013 Beijing Anzhen Hospital database. Comparators: Comparators were the rest of the patients in the study who presented for AF ablation in the absence of structural heart or systemic disease including RA. Follow‐up time was 1 year.
Number of subjects	Cases: 107 Comparators: 428	Cases: 45 Comparators: 45	Cases: 15 Comparators: 60
Baseline characteristics of subjects	Age, year: Cases: 64 ± 10 Comparators: 65 ± 10 Female: Case: 48.6% Comparators: 43.9% Median AF duration, year (IQR): Case: 2.0 (0.75, 5.0) Comparators: 2.3 (0.6, 5.3) AD subgroup: CTD: 76.6% OD: 23.4% Single AD: 86.0% Multiple AD: 14.0% AD subtype: RA: 38.3% AS: 21.5% Hashimoto thyroiditis: 15.9% Sjogren syndrome: 14.0% Psoriasis: 12.1% Autoimmune hepatitis: 4.7% Grave disease: 3.7% Multiple arteritis: 0.9% SLE: 0.9% Systemic sclerosis: 0.9% Polymyalgia rheumatica: 0.9% Median AD duration, year (IQR): CTD: 10.0 (5.0, 20.0) OD: 10.0 (4.0, 15.5) Single AD: 10.0 (5.0, 20.0) Multiple AD: 12.0 (10.0, 20.0) PsAF: Case: 34.6% Comparators: 36.7% HTN: Cases: 52.3% Comparators: 57.7% DM: Case: 16.8% Comparators: 18.9% Stroke: Case: 7.5% Comparators: 7.9% LAD, mm: Case: 38.8 ± 5.2 Comparators: 39.7 ± 5.6 LVEF, %: Case: 63.1 ± 6.0 Comparators: 63.5 ± 7.6 DMARD: Case: 19.6% Comparators: 0% Corticosteroid: Case: 15.9% Comparators: 0% HF: Case: 9.3% Comparators: 10.3% CAD: Case: 21.5% Comparators: 22.2% PAD: Case: 5.6% Comparators: 7.9% Hyperlipidemia: Case: 39.6% Comparators: 38.8% CKD: Case: 5.6% Comparators: 7.9% EHRA EHRA I: Case: 1.9% Comparators: 8.2% EHRA II: Case: 45.8% Comparators: 58.9% EHRA III: Case: 52.3% Comparators: 31.8% EHRA IV: Case: 0% Comparators: 0.4% sPAP, mmHg: Case: 32.6 ± 7.3 Comparators: 30.3 ± 7.5 mPAP, mmHg: Case: 22.0 ± 4.8 Comparators: 20.5 ± 5.1	Age, year: Cases: 66.3 ± 7.7 Comparators: 68.0 ± 7.3 Female: Case: 66.7% Comparators: 66.7% Duration of AF, year: Case: 3.67 ± 3.82 Comparators: 4.66 ± 4.95 PAF: Case: 60% Comparators: 62% PsAF: Case: 36% Comparators: 38% Long‐standing PsAF: Case: 4% Comparators: 0% CHA_2_DS_2_VASc score: Case: 2.73 ± 1.80 Comparators: 3.18 ± 2.05 HAS‐BLED score: Case: 3.47 ± 1.24 Comparators: 3.73 ± 1.50 LA volume index, mL/m^2^: Case: 38.0 ± 10.3 Comparators: 38.6 ± 12.3 LVEF, %: Case: 57.9 ± 8.37 Comparators: 58.7 ± 5.92 Immunosuppressive agents: Case: 38% Comparators: 0% Steroids: Cases: 13% Comparators: 0% AAD: Case: 44% Comparators: 40% CRP, mg/L: Case: 5 (3.0–11.5) Comparators: 2 (0.7–3.0) ESR, mm/1 h: Case: 10 (3.0–23.0) Comparators: 3.5 (3.0–6.0) Valvular heart disease: Case: 24% Comparators: 16% BMI, kg/m^2^: Case: 30.6 ± 5.44 Comparators: 31.0 ± 6.78	Age, year: Cases: 63.67 ± 8.02 Comparators: 63.57 ± 9.19 Female: Case: 80.0% Comparators: 80.0% AF duration, year: Case: 5.67 ± 5.19 Comparators: 5.03 ± 5.22 PAF: Case: 60% Comparators: 60% PsAF: Case: 40% Comparators: 40% Hypertension: Cases: 53.33% Comparators: 61.67% Diabetes mellitus: Case: 6.67% Comparators: 8.33% Stroke: Case: 20% Comparators: 25% LAD, mm: Case: 38.83 ± 6.92 Comparators: 39.29 ± 7.16 LVEF, %: Case: 63.13 ± 6.79 Comparators: 63.06 ± 9.25 Immunosuppressive agents: Case: 13.33% Comparators: 0% Methylprednisolone: Case: 26.67% Comparators: 0% Statin: Case: 33.3% Comparators: 26.67% CRP: Case: 4.14 ± 2.30 Comparators: 1.81 ± 2.35 Body temperature, ⁰C: Case: 36.3 ± 0.1 Comparators: 6.3 ± 0.2 White blood cell count,/mm^3^: Case: 6361 ± 1567 Comparators: 5632 ± 1200 Neutrophils,/mm^3^: Case: 3949 ± 1461 Comparators: 3308 ± 973 Lymphocytes,/mm^3^: Case: 1963 ± 557 Comparators: 1867 ± 525 Monocytes,/mm^3^: Case: 337 ± 147 Comparators: 309 ± 102 Neutrophil/lymphocyte ratio: Case: 2.16 ± 1.23 Comparators: 1.94 ± 0.94 Hemoglobin, g/dL: Case: 12.6 ± 1.8 Comparators: 13.1 ± 1.7 Platelet count, x10 3: Case: 196 ± 59 Comparators: 198 ± 44
Diagnosis of AF	AF was detected by 12 leads ECG, pacemaker, ICD electrocardiogram, or Holter ECG.	AF was detected by 12‐lead ECG and Holter monitoring.	AF was detected by ECG or Holter monitoring.
Ablation strategy	PVI, lines, CTI, non‐PV triggers, CFAE Cases: 100% PVI, 59.8% CTI, 64.5% linear ablation, 18.7% non‐PV trigger, 9.3% CFAE Comparators: 100% PVI, 59.5% CTI, 62.4% linear ablation, 8.4% non‐PV trigger, 4.9% CFAE	PVI, lines, CTI Cases: 36% PVI, 13% PVI + lines, 31% PVI + CTI, 20% PVI + lines + CTI Comparators: 33% PVI, 16% PVI + lines, 38% PVI + CTI, 13% PVI + lines + CTI	PVI, lines, CTI
Procedure‐related complication	Case: 0.9% cardiac tamponade, 0.9% postcardiac injury syndrome Comparators: 0.2% cardiac tamponade 0.9% pseudoaneurysm 0.6% arterio‐venous fistula No periprocedural thromboembolism	Case: 4.4% groin hematoma Comparators: 2.2% groin hematoma No periprocedural thromboembolism	Case: 6.7% groin hematoma Comparators: none No periprocedural thromboembolism
Diagnosis of arrhythmic recurrence	The diagnosis of AF or AT recurrence was supported by 12 lead ECG and TTE on the day after the procedure, 24‐h ECG at 1, 2, 3, and 6 months and incident ECG. Cases: 41.1% (AD), 46.3% (CTD) Comparators: 36.2% AF recurrence within 3 months postablation defined as early recurrence. Cases: 36.4% (AD) Comparators: 13.5%	The diagnosis of AF recurrence was supported by chart review and diagnostic testing performed at follow‐up visits at 3 months and 1 year. AF recurrence was defined as documented AF on 12‐lead ECG and Holter monitoring. Cases: 44% Comparators: 18% AF recurrence within 3 months postablation defined as early recurrence. Cases: 33% Comparators: 13%	The diagnosis of atrial tachyarrhythmia (AF, AFL, or AT) was supported by ECG or Holter monitoring at 1, 3, 6, 12 months, and when experienced symptoms. Telephone interviews were also conducted monthly. Cases: 33.3% Comparators: 31.7% AF recurrence within 3 months postablation defined as early recurrence. Cases: 33.3% Comparators: 15.0%
Redo ablation procedure	Cases: 32.7% (82.8% PV gaps, 77.1% linear ablation, 25.7% non‐PV trigger, 11.4% CFAE) Comparators: 9.3% (95.0% PV gaps, 55.0% linear ablation, 12.5% non‐PV trigger, 7.5% CFAE)	Cases: 13% (50% PVI, 17% PVI + lines, 33% PVI + CTI) Comparators: 2% (100% PVI)	Cases: 20% (100.0% PVI, 66.7% surgical ablation) Comparators: NA
Confounder adjusted in the multivariate analysis	Age, gender, AF type, duration of AF history, and comorbidities (stroke, HF, HTN, CAD, PAD, hyperlipidemia, CKD, and DM)	Age, gender, history of HF, DM, transient ischemic attack/stroke, HTN, peripheral vascular disease, hyperlipidemia, and CAD	Age, gender, AF type, AF duration, LAD, LVEF, HTN, RA, WBC count, neutrophil count, lymphocyte count, NLR, monocyte count, and CRP level
Newcastle‐Ottawa score	Selection: 3 stars Comparability: 2 stars Outcome: 3 stars	Selection: 4 stars Comparability: 2 stars Outcome: 3 stars	Selection: 4 stars Comparability: 2 stars Outcome: 3 stars

Abbreviations: AAD, antiarrhythmic drug; AD, autoimmune disease; AF, atrial fibrillation; AFL, atrial flutter; anti‐CCP, anticyclic citrullinated peptide antibodies; AT, atrial tachycardia; BMI, body mass index; CA, catheter ablation; CAD, coronary artery disease; CFAE, complex fractionated atrial electrograms; CKD, chronic kidney disease; CRP, C‐reactive protein; CTI, cavotricuspid isthmus; DM, diabetes mellitus; DMARD, disease‐modifying antirheumatic drugs; ECG, electrocardiogram; EF, ejection fraction; eGFR 5 estimated glomerular filtration rate; EHRA, European Heart Rhythm Association symptom classification; ESR, erythrocyte sedimentation rate; HF, heart failure; HTN, hypertension; ICD, intracardiac defibrillator; IQR, interquartile range; LA, left atrium; LAD, left atrium diameter; LV, left ventricle; LVEF, left ventricular ejection fraction; MI, mitral isthmus; mPAP, mean pulmonary artery pressure; NA, not available; NLR, neutrophil/lymphocyte ratio; NYHA, New York Heart Association Class; PAD, peripheral artery disease; PAF, paroxysmal atrial fibrillation; PV, pulmonary vein; PVI, pulmonary vein isolation; PsAF, persistent atrial fibrillation; RA, rheumatoid arthritis; RF, rheumatoid factor. RL, roofline; sPAP, systolic pulmonary artery pressure; TTE, transthoracic echocardiography.

### 3.1 Risk of AF Recurrence After CA Among Patients With RA

**Figure 2:**
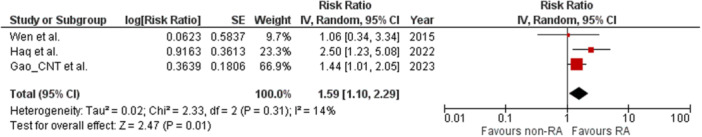
Forest plot of the meta‐analysis for AF recurrence after index catheter ablation for AF.

A total of three cohort studies [9, 10, 11] reported the risk of AF recurrence after CA between patients with and without RA. The pooled analysis found a significantly increased risk of AF recurrence after CA among patients with RA compared to patients without RA with the pooled risk ratio of 1.59 (95% CI, 1.10–2.29). The between‐study heterogeneity was insignificant with an *I* ^2^ of 14%. Figure 2 demonstrates the forest plot of this meta‐analysis.

### 3.2 Risk of Early AF Recurrence After CA Among Patients with RA

**Figure 3:**
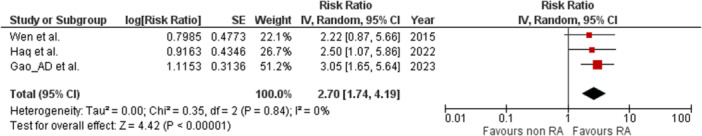
Forest plot of the meta‐analysis for early AF recurrence after index catheter ablation for AF.

A total of three cohort studies [9, 10, 11] reported the risk of early AF recurrence after CA between patients with and without RA. The pooled analysis found a significantly increased risk of early AF recurrence after CA among patients with RA compared to patients without RA with the pooled risk ratio of 2.70 (95% CI, 1.74–4.19). The between‐study heterogeneity was insignificant with an *I* ^2^ of 0%. Figure 3 demonstrates the forest plot of this meta‐analysis.

### 3.3 Evaluation for Publication Bias

Funnel plot for evaluation of publication bias was not created due to insufficient number of included studies.

## 4 Discussion

The current systematic review and meta‐analysis is the first comprehensive study to summarize data from all available cohort studies to determine the risk of AF recurrence after CA among patients with RA. Our data show a 1.59‐fold higher risk of AF recurrence and a 2.70‐fold higher risk of early AF recurrence after CA compared to patients without RA. Potential explanations for the observed increased risk are discussed below.

First, systemic inflammation is a known risk factor for the occurrence and development of AF as a result of atrial structural and electrical remodeling, impacting AF onset and maintenance [15]. Pro‐inflammatory marker such as C‐reactive protein (CRP) is significantly elevated in new‐onset AF [16] and recurrent AF postablation [17] or postcardioversion [18]. Tumor necrosis factor (TNF) and platelet‐derived growth factor‐A (PDGF‐A) play a key role in electrical remodeling. TNF alters calcium handling [19] and PDGF‐A shortens action potentials [20]. Inflammation also affects conduction, enhancing heterogeneity via altered connexin expression, potentially forming the basis for AF development [21]. Therefore, it is conceivable that individuals with high inflammatory burden, such as those with RA, would have a higher tendency for development and recurrence of AF.

Interestingly, the pooled analysis also found an increased likelihood of early AF recurrence (within 90 days postablation) in RA patients. This could potentially be caused by more immediate inflammatory response induced by CA in patients with RA [22] as the procedure tends to be more complex compared to patients without RA [10]. Early recurrence within the first 90 days overlaps with the traditional blanking window, where AF episodes aren't typically seen as indicators of long‐term recurrence. However, their prognostic value remains significant [23]. All included studies did not apply a blanking period and found that RA patients were more likely to develop early recurrence compared to controls. Notably, in both groups from Wen et al., [11] none of the early recurrent AF episodes (within 90 days postablation) spontaneously resolved during follow‐up.

Despite the evidence supporting the role of inflammation in pathogenesis of AF, benefit of treatment of inflammation remains unclear. A randomized controlled‐trial suggested that administering corticosteroids during electrical cardioversion may reduce AF recurrence [24]. However, subsequent meta‐analysis suggested that use of immunosuppression and/or corticosteroids during ablation was associated with a decrease in early recurrence of AF, but not late recurrence [25]. Some evidence suggests that statins, with their anti‐inflammatory effects, may reduce AF recurrence postablation. A meta‐analysis by Liu et al. [26] found that statin therapy significantly reduced AF recurrence risk, likely due to reduced oxidative stress and inflammation. However, outcomes vary by patient population, timing, and therapy type, making the evidence inconclusive. Further research is needed to fully understand the relationship between inflammation modulation and AF recurrence after ablation.

The second potential explanation is related to the potential role autoimmunity. In a study by Haq et al., [9] six RA patients who underwent repeated ablation within a year of the initial procedure exhibited conduction gaps between the pulmonary veins and the left atrium. Among these patients, four showed a positive autoantibody titer shortly after the initial ablation, suggesting that altered immune response in RA may contribute to the occurrence of the conduction gaps. Additionally, RA patients had significantly higher levels of inflammatory markers, such as ESR and CRP, before CA [9, 11]. It is important to note that additional autoimmune markers, such as matrix metalloprotease (MMP), were not explored, and the studies lacked consistent data on RA activity (e.g., DAS28 scores) at the time of AF ablation. This absence limits our ability to directly correlate RA activity with AF recurrence risk. However, RA activity could significantly influence AF recurrence by exacerbating atrial remodeling and arrhythmic risk, highlighting the need for further research in this area.

The third explanation is related to common predisposing factors to both RA and AF. Previous reports demonstrated higher prevalence of obstructive sleep apnea (OSA) and congestive heart failure (CHF) in RA patients compared to the general population. [27, 28]. Given that OSA and CHF are significant risk factors for AF recurrence [29, 30], the observed increased risk could be at least partially explained by these underlying conditions.

It should be noted that baseline characteristics such as AF duration, type, left atrial size, and comorbidities are shown in Table 1. However, detailed data comparing AF complexity between RA patients and controls were not uniformly available, making it difficult to fully assess whether increased AF recurrence in RA patients is linked to greater baseline AF complexity. Chronic inflammation in RA may lead to more extensive atrial remodeling and fibrosis, suggesting a more complex atrial substrate in RA patients, potentially driving higher recurrence rates. This highlights the need for future research with more detailed AF characteristics.

The results of our study, demonstrating an increased risk of AF recurrence after CA in patients with RA, have significant clinical implications. We focused on RA rather than multiple autoimmune conditions due to its well‐established link with systemic inflammation and CVD, making it particularly relevant to study in the context of AF recurrence postablation. Given the chronic inflammatory nature of RA, our findings suggest that these patients may require more intensive monitoring and management after AF ablation. Clinicians should consider the increased risk when developing postablation care plans for RA patients, which may include closer follow‐up, additional anti‐inflammatory therapies, or alternative ablation strategies tailored to this population. Furthermore, the study highlights the importance of managing systemic inflammation in RA patients as part of a comprehensive approach to reduce AF recurrence and improve long‐term outcomes. By integrating these findings into clinical practice, healthcare providers can better stratify risk and potentially improve the success rates of AF ablation in patients with RA.

While the studies included in this analysis were of good quality, and the literature review process was comprehensive, we acknowledge some limitations that might jeopardize the validity of the results. First, variations in study design, participant characteristics, and ablation strategies may limit the interpretability of the pooled result, although between‐study statistical heterogeneity was insignificant. Second, the generalizability of the findings to other populations could be limited as only one included study was conducted in the United States [9] and the other two were from China [10, 11]. Lastly, an assessment of publication bias was not feasible due to the restricted number of included studies. Consequently, the presence of publication bias in this meta‐analysis remains unknown.

## 5 Conclusion

The current study found that patients with RA have a higher risk of AF recurrence after CA for AF. Further investigations are needed to determine whether this association is causal and how it should be addressed in clinical practice.

## Author Contributions

All authors designed the study. Pongprueth Rujirachun, Phuuwadith Wattanachayakul, and Svita Taveeamornrat collected the data and drafted the manuscript. Pongprueth Rujirachun and Patompong Ungprasert performed the statistical analysis. Pongprueth Rujirachun, Nithi Tokavanich, and Krit Jongnarangsin made critical revisions. Pongprueth Rujirachun, Patompong Ungprasert, and Krit Jongnarangsin revised the final manuscript. All authors read and approved the final manuscript.

## Ethics Statement

This study is a meta‐analysis, which involves synthesizing and analyzing data from previously published studies. Since no new data were collected directly from patients or participants, and the data used were from studies that had already received ethical approval, obtaining additional ethical approval and patient consent was not required. Thus, this section is not applicable.

## Conflicts of Interest

The authors declare no conflicts of interest.

## Supporting information

Supporting information.

Supporting information.

## Data Availability

Data sharing not applicable to this article as no datasets were generated or analyzed during the current study. The data that supports the findings of this study is available on request from the corresponding author.
