# Supplementary material for: Atrial Fibrillation Recurrence Risk After Catheter Ablation in Patients With Rheumatoid Arthritis: A Systematic Review and Meta‐Analysis
Source: Clin Cardiol. 2025 Jan 16;48(1):e70021. doi: 10.1002/clc.70021 (PMC11738958; doi:10.1002/clc.70021)
Supplement: Supplementary file 1 — Supporting information. [file CLC-48-e70021-s001.docx]

**Supplementary data 1**

Search strategy

**EMBASE**

1. 'atrial fibrillation'/exp OR 'atrial fibrillation'
2. 'atrial tachyarrhythmia'/exp OR 'atrial tachyarrhythmia'
3. 'heart atrium arrhythmia'/exp OR 'heart atrium arrhythmia'
4. 'supraventricular tachycardia'/exp OR 'supraventricular tachycardia'
5. 'catheter ablation'/exp OR 'catheter ablation'
6. 'pulmonary vein isolation'/exp OR 'pulmonary vein isolation'
7. 'rheumatoid arthritis'/exp OR 'rheumatoid arthritis'
8. 'inflammatory arthropathy'/exp OR 'inflammatory arthropathy'
9. 'autoimmune disease'/exp OR 'autoimmune disease'
10. 'connective tissue disease'/exp OR 'connective tissue disease'
11. #1 OR #2 OR #3 OR #4
12. #5 OR #6
13. #7 OR #8 OR #9 OR #10
14. #11 AND #12 AND #13

**Medline**

1. exp Atrial Fibrillation/ or Atrial Fibrillation.mp.
2. Atrial arrhythmia.mp.
3. Atrial tachyarrhythmia.mp.
4. exp Atrial Flutter/ or Atrial Flutter.mp.
5. exp Tachycardia, Ectopic Atrial/ or Tachycardia, Ectopic Atrial.mp.
6. exp Tachycardia, Supraventricular/ or Tachycardia, Supraventricular.mp.
7. exp Catheter Ablation/ or catheter ablation.mp.
8. PVI.mp.
9. Pulmonary vein isolation.mp.
10. rheumatoid arthritis.mp. or exp Arthritis, Rheumatoid/
11. autoimmune disease.mp. or exp Autoimmune Diseases/
12. inflammatory arthropathy.mp.
13. connective tissue disease.mp. or exp Connective Tissue Diseases/
14. or/1-6
15. or/7-9
16. or/10-13
17. 14 and 15 and 16
